# Reduced Retinal Function in the Absence of Na_v_1.6

**DOI:** 10.1371/journal.pone.0031476

**Published:** 2012-02-15

**Authors:** Benjamin J. Smith, Patrice D. Côté

**Affiliations:** 1 Department of Biology, Dalhousie University, Halifax, Nova Scotia, Canada; 2 Department of Ophthalmology and Visual Sciences, Dalhousie University, Halifax, Nova Scotia, Canada; Dalhousie University, Canada

## Abstract

**Background:**

Mice with a function-blocking mutation in the *Scn8a* gene that encodes Na_v_1.6, a voltage-gated sodium channel (VGSC) isoform normally found in several types of retinal neurons, have previously been found to display a profoundly abnormal dark adapted flash electroretinogram. However the retinal function of these mice in light adapted conditions has not been studied.

**Methodology/Principal Findings:**

In the present report we reveal that during light adaptation these animals are shown to have electroretinograms with significant decreases in the amplitude of the a- and b-waves. The percent decrease in the a- and b-waves substantially exceeds the acute effect of VGSC block by tetrodotoxin in control littermates. Intravitreal injection of CoCl_2_ or CNQX to isolate the a-wave contributions of the photoreceptors in littermates revealed that at high background luminance the cone-isolated component of the a-wave is of the same amplitude as the a-wave of mutants.

**Conclusions/Significance:**

Our results indicate that *Scn8a* mutant mice have reduced function in both rod and the cone retinal pathways. The extent of the reduction in the cone pathway, as quantified using the ERG b-wave, exceeds the reduction seen in control littermates after application of TTX, suggesting that a defect in cone photoreceptors contributes to the reduction. Unless the postreceptoral component of the a-wave is increased in *Scn8a* mutant mice, the reduction in the b-wave is larger than can be accounted for by reduced photoreceptor function alone. Our data suggests that the reduction in the light adapted ERG of *Scn8a* mutant mice is caused by a combination of reduced cone photoreceptor function and reduced depolarization of cone ON bipolar cells. This raises the possibility that Na_v_1.6 augments signaling in cone bipolar cells.

## Introduction

Mice with a null mutation in *Scn8a*, which encodes the voltage-gated sodium channel (VGSC) Na_v_1.6, show a severe physiological defect in dark adapted, or scotopic, vision although morphologically the retina appears unchanged [1]. Outer retinal function in these mice is similar to younger animals suggesting that Na_v_1.6 is necessary for maturation of the rod, and possibly, cone photoreceptors. The effect of loss of function mutations in Na_v_1.6 in light adapted conditions has not yet been tested. Development and maturation of rod and cone photoreceptors is governed by different factors [2], [3] suggesting the possibility of a relatively intact cone system despite a substantial loss in rod function in *Scn8a*-null mice.

Loss of Na_v_1.6 could cause cone photoreceptor dysfunction but may also alter the light adapted ERG in additional ways. There are components of the light adapted electroretinogram (ERG) that are sensitive to tetrodotoxin (TTX), the canonical VGSC blocker. Reduction in these components in *Scn8a*-null mice could help define the molecular identity of the VGSC isoforms contributing to signal processing in cone bipolar cells. VGSCs are involved in cone bipolar cell signaling in several animal models including rat, goldfish, salamander, and human [4]–[9] where they seem to be involved in increasing the gain in the cone bipolar cells that depolarize in response to a light flash (ON bipolar cells) under dim conditions [6]. In support of this model TTX in rodents results in reduces the photopic b-wave (an ERG waveform mainly generated in light adapted conditions by the ON cone bipolar cells in mice and rats) amplitude [10]–[12], an effect that is greatest under dark adapted conditions and lessens as the retina light adapts [13]. Additionally, TTX affects the photopic negative response (PhNR), a corneal negative response following the photopic b-wave [13], [14] believed to be generated by tertiary retinal neurons.

We report that the ERG of *Scn8a*-null mice is profoundly reduced at a range of light intensities, with significant losses in both the a- and b-waves. The reduction in b-wave is far more profound than the response to TTX application in control littermates, therefore loss of Na_v_1.6 channel amplification of ON cone bipolar cell signal is an insufficient explanation. Although the a-wave of control littermates includes a substantial postreceptoral response it is not affected by TTX, suggesting that the a-wave reduction in mutants is due to a defect in cone function. We suggest that the mechanism that leads to delayed maturation of the rod acts on the cones as well, although this does not exclude the possibility of involvement of Na_v_1.6 in amplification of cone ON bipolar cell signaling.

## Methods

### 1. Electroretinography

All animal procedures were completed in accordance to animal care guidelines established by the Canadian Council on Animal Care and in accordance with the ARVO statement for the Use of Animals in Ophthalmic and Vision Research. Mice were housed under a 12 hour light/dark cycle with free access to food and water.

*Scn8a* ^dmu^ mice, which harbor a function-blocking mutation in the *Scn8a* gene, have been described previously [1], [15]. The mutation was found to consist of a single nucleotide deletion in the sequence coding for the first interdomain loop of Na_v_1.6. The resulting frameshift in the open reading frame results in the presence of a stop codon a short distance downstream of the mutation. The resulting neurological phenotype consists in ataxia appearing at P12–14, followed by paralysis of the hindquarters, muscle wasting and death at approximately P23–25. Thus, we have chosen to perform ERGs at P16 because at that age recordings can be reliably obtained. Homozygous 16 day old *Scn8a* ^dmu^ and wild-type littermates were dark adapted for at least 4 hours before being anesthetized under dim red light by intraperitoneal injection of Avertin (2,2,2 Tribromoethanol, Sigma Aldrich, St-Louis, MO) dissolved in amylene hydrate (tertiary amyl alcohol, 275 mg/kg, Sigma Aldrich). The pupils were dilated with the mydriatic agent cyclopenolate HCl 0.5% (Alcon, Fort-Worth, TX) and a 0.5% proparacaine hydrocloride (Alcon) was applied as a topical analgesic to reduce eye movement and irritation. Final pupil diameter was approximately 1.3 mm in both mutants and controls. Body temperature was maintained at 37°C with a heated pad and monitored rectally. Mice were sacrificed by anesthetic overdose followed by cervical dislocation and tails were collected to confirm genotype using a previously described protocol [1]. The active electrode was a Dawson-Trick-Litzkow-plus microconductive fiber (Diagnosys, Littleton, MA) placed on the corneal surface and hydrated with 2.5% hydroxypropyl methylcellulose solution to maintain conductivity. Platinum subdermal electrodes (Grass Instruments, Quincy, Mass, IL) were placed in the base of the nose (reference) and in the tail (ground). Signal from the corneal electrodes was amplified 10,000 fold using a differential amplifier with a bandwidth of 3–1000 Hz (P511, Grass instruments). An A/D instrument converter (GW Instruments, Summerville, SC) was used to digitize three hundred sample points at a rate of 1000 Hz.

Mice were initially examined in the dark-adapted condition to ensure proper corneal contact of the electrode using a PS3 photostimulator (Grass Instruments). A series of flashes with increasing energy from −2.6 to 1 log cd⋅s/m^2^ in 0.4 or 0.6 log unit steps (Kodak Wratten no. 96 neutral density filters) depending on background luminance were delivered in a Ganzfield stimulator (LKC Technologies). Background illumination started at −0.5 log cd/m^2^ to a maximum of 2.1 log cd/m^2^. All luminance measurements are in photopic cd/m^2^. Mice were monitored while exposed to a series of 5 flashes every 5 minutes following exposure to a new background luminance until the response stabilized.

### 2. Data Analysis

Analysis of ERG waveforms was completed using a custom ERG analysis toolbox written for Matlab (Mathworks, Natick, MA) by Dr. Francois Tremblay and Matt Boardman. The amplitude of the a-wave was measured as the difference between the baseline and the response at 18 ms post-stimulus and b-wave amplitude was measured from a-wave trough b-wave peak following the trough. Comparison of a- and b-wave amplitudes was carried out using Microsoft (Redmond, WA) Excel.

### 3. Intravitreal injection

Following application of a topical anesthetic to the cornea of anesthetized mice 0.5 µl of 20 mM cobalt chloride (CoCl_2_) (Sigma Aldrich), 20 uM TTX, or 1 mM 6-Cyano-7-nitroquinoxaline-2,3-dion (CNQX), dissolved in phosphate buffered saline pH 7.4 or, or vehicle alone was injected into the vitreous via a 30-gauge needle mounted on a Hamilton syringe attached to a micromanipulator under dim red light. The injection site was located approximately 0.5 mm behind the ora serrate. Following injection the mice were dark adapted for approximately 30 minutes to allow for diffusion of the drug before recording.

### 4. Immunohistochemistry

Following anesthetic overdose and cervical dislocation, eyes were enucleated and hemisected. Retinas were carefully removed from eyecups in cold PBS (pH 7.3), quartered, and immersion fixed in 4% paraformaldehyde for 12 hours at 4°C. The retina was blocked with 2.5% BSA in PBS for 1 hour. Peanut agglutinin (PNA) lectin (FITC-conjugated lectin 0.2 mg/mL from *Arachis hypogaea*; Sigma Aldrich) was dissolved in the blocking solution at 1∶100 dilution and added to the retina to stain for cone photoreceptors. After 12 hours of incubation in PNA lectin, the retina was washed four times in PBS (20 minutes each) and mounted using Mowiol 4–88 antifade mounting medium (Calbiochem, San Diego, CA) and a glass coverslip. The samples were imaged *en face* using a Nikon Eclipse 90i fluorescence compound microscope equipped with an FITC filter set, a DXM 1200c digital camera and NIS-Elements image analysis software.

## Results

**Figure 1:**
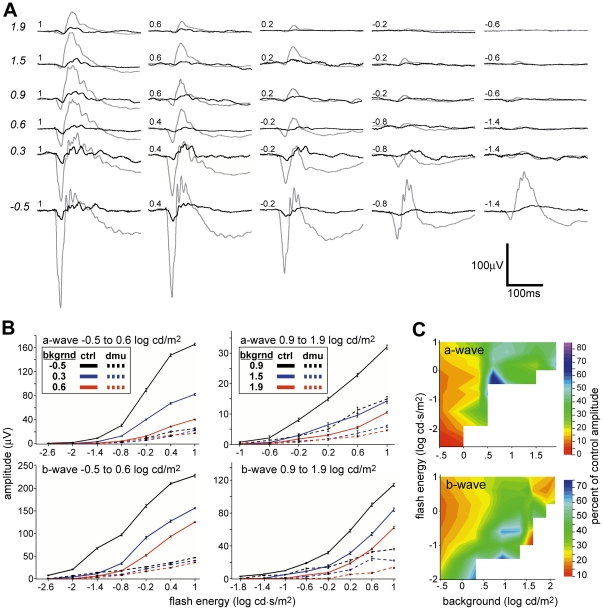
The a- and b-waves of *Scn8a* ^dmu^ mice is reduced under nearly all stimulus and background conditions. (a) Intensity series comparing representative *Scn8a* ^dmu^ (black) and control littermate (grey) ERGs over background luminances increasing from −0.5 to 1.9 log cd/m^2^ (italicized) with a flash energies from −2.6 to 1 log cd·s/m^2^. (b) Intensity response graphs for *Scn8a* ^dmu^ and control littermate a- and b-waves for backgrounds −0.5, 0.3, 0.6 0.9, 1.5, 1.9 log cd/m^2^. (c) Isocline representation of the a-wave (top) and b-wave (bottom) in *Scn8a* ^dmu^ mice as the percent of control responses shown as a function of flash energy and background. Variance in isocline representation is equal to that of the intensity response curves in panel (b).

A recent study by Mojumder et al. [13] shows that the effects of TTX on the b-wave of the adult rat ERG are dependent on background illumination, indicating that the amplifying effects of VGSCs on ON cone bipolar cells are best examined with an adapting field that varies over a range of background illumination. A typical series of responses to a bright flash (Fig. 1a, b) over a range of flash intensities and adapting backgrounds revealed that *Scn8a* ^dmu^ a- and b-wave amplitudes were reduced relative to controls in all stimulus conditions and background conditions.

A comparison of a-wave amplitude between *Scn8a* ^dmu^ mice (n = 16) and wild-type littermates (n = 16) shows that the a-wave of *Scn8a* ^dmu^ mice is significantly reduced under nearly all stimulus and background conditions (*p*≤0.001 for the flash energies 1-0.6 log cd s/m^2^ on background 1.9 log cd/m^2^, and flash energies −0.2 to 1 log cd/m^2^ on backgrounds −0.5 to 1.5 log cd/m^2^). We plotted the a-wave amplitude of *Scn8a* ^dmu^ mice normalized to the control response as a function of both background luminance and flash energy (Fig. 1c top) this showed that the percent difference is similar (around 40% of controls) at higher background luminance and does not vary strongly with flash energy. However we saw a trend in the 3 dimmest backgrounds where the a-wave of *Scn8a* ^dmu^ mice was most strongly reduced under the dimmest background condition (16% of control for −0.5 log cd s/m^2^).

The b-wave of *Scn8a* ^dmu^ mice is significantly reduced under almost all stimulus and background conditions as well (Fig. 1a, b; *p*≤0.001 for the flash energy 0.2–1 log cd s/m^2^ on backgrounds 1.5–1.9 log cd/m^2^, flash energies −0.2–1 log cd⋅s/m^2^ on backgrounds 0.9–1.5 log cd/m^2^, and flash intensities −0.8–1 log cd⋅s/m^2^ on backgrounds −0.5 to 0.6 log cd/m^2^), non-significant differences were seen occasionally with low energy flashes in the middle background luminances (0.6–1.5 log cd/m^2^). When analyzed as the percent difference from controls, at the brightest flashes the b-wave is most reduced in the brightest and dimmest backgrounds with less reduction in the middle backgrounds (Fig. 1c bottom).

**Figure 2:**
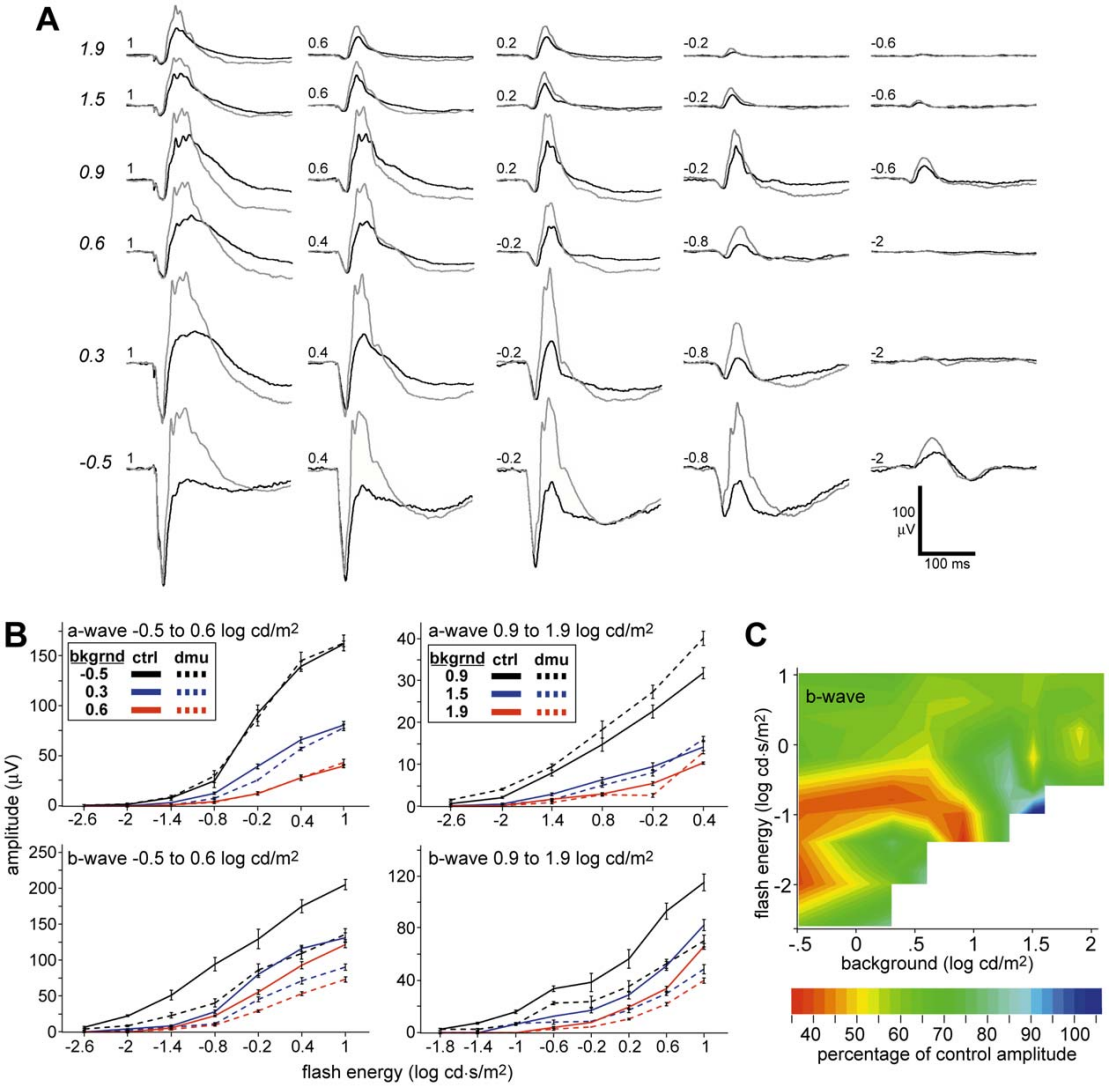
TTX does not affect the a-wave and the effect on the b-wave is most pronounced at lower stimulus intensity and background luminance. (a) Intensity series comparing representative TTX-injected (black) and sham-injected control eye (grey) ERGs over background luminance increasing from −0.5 to 1.9 log cd/m^2^ with a flash energies ranging from −2.6 to 1 log cd·s/m^2^. (b) Intensity response graphs for TTX a and b-waves for backgrounds −0.5, 0.3, 0.6 log cd/m^2^ (dim) and 0.9, 1.5, 1.9 log cd/m^2^ (bright) (c) Isocline representation of the b-wave amplitude in TTX treated wild-type eyes mice as the percent of averaged control responses shown as a function of flash energy and background. Variance in isocline representation is equal to that of the intensity response curves in panel (b).

We injected TTX intravitreally in postnatal day (P) 16 wild-type controls (n = 5) to quantify the contribution that VGSCs make to the b-wave of juvenile mice (Fig. 2a, b). TTX reduced the b-wave by roughly 40% averaged across backgrounds (Fig. 2c). (*p*≤0.01 for the stimulus intensities 1 to −2 log cd⋅s/m^2^ on background −0.5 to 0.9 log cd/m^2^, and flash energies 1- to −1.4 log cd/m^2^ on backgrounds −0.9 to 1.9 log cd/m^2^ *p*≥0.05 for all other stimulus intensities and backgrounds). We found that in general there was little change in the effect of TTX measured as the percent difference from the control eye as we increased the background luminance with a bright stimulus, suggesting some differences with Mojumder et al. [13] probably due either to the immature state of the retina, species differences, or differences in stimuli. There was a stronger effect of flash energy over a single background especially with dimmest 4 backgrounds, such that the effects of TTX on the b-wave were most substantial in response to a dim flash presented on a dim adapting background. Injection of TTX had no significant effect on the amplitude of the a-wave under any combination of background and stimuli.

**Figure 3:**
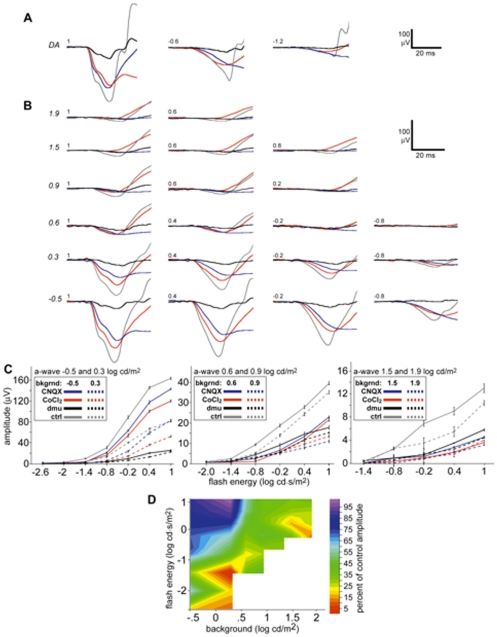
At high background luminances, the amplitude of the cone-isolated component of the a-wave of controls is similar to the a-wave of *Scn8a* ^dmu^ mice. (a) Intensity series comparing a-wave amplitudes of *Scn8a* ^dmu^ mice (black) to control a-waves (grey) and control a-waves in the presence of either CNQX (red) or cobalt (blue) for dark adapted (DA) conditions. (b) Intensity series in light adapting conditions. (c) Intensity response graphs for all four conditions in dim (−0.5 and 0.3 log cd/m^2^), medium (0.6 and 0.9 log cd/m^2^) and bright (1.5 and 1.9 log cd/m^2^) backgrounds (italicized). (d) Isocline representation of the CNQX treated a-wave in as the percent of control responses shown as a function of flash energy and background. Variance in isocline representation is equal to that of the intensity response curves in panel (b).

We found that even under a series of backgrounds that should desensitize rods (1.3–2.1 log cd/m^2^) a small a-wave remained in control mice. Previous work in adult mice [16] has demonstrated that the adult photopic a-wave is generated by a combination of a small photoreceptor component in combination with substantial negative response generated by post-receptoral cells. We isolated the photoreceptor contribution to the a-wave of control littermates using both CNQX (n = 5) and CoCl_2_ (n = 4) to compare to the a-wave of *Scn8a* ^dmu^ mice (Fig. 3a–c). The percentage of the a-wave generated by post-receptoral cells increased as the background increased for both conditions, increasing rapidly around 0.9 log cd/m^2^. The a-wave was reduced more strongly in response to CNQX, probably because of the intrusion of the b-wave on the a-wave, which was absent in CoCl_2_ treated retinas. We found that in P16 mice injection of either CoCl_2_ or CNQX substantially reduced the scotopic a-wave, an unexpected result, given that the dark adapted a-wave is generally considered to be generated mainly by the photoreceptors, but see Dang *et al.* [17]. This reduction may be due to the immature state of the retina, a possibility we are continuing to investigate. It should be noted that neither TTX nor sham injections of PBS (pH 7.4) reduced the amplitude of the scotopic a-wave in controls. The amplitude of the isolated photoreceptor component of the a-wave in littermates closely matched the amplitude of the *Scn8a* ^dmu^ a-wave for the brightest 6 backgrounds with rapid divergence for the dimmest backgrounds and in scotopic conditions.

**Figure 4:**
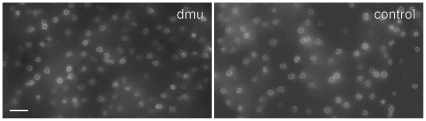
Cones are present at normal density in *Scn8a* ^dmu^ mice as indicated by FITC-conjugated peanut agglutinin lectin staining in *Scn8a* ^dmu^ homozygous mice (dmu) and wild-type littermates (control). Bar = 10 µm.

The a-wave amplitude was reduced in *Scn8a* ^dmu^ mice in photopic conditions, an indication of reduced cone phototransduction. Although previous results have shown normal cone presence using retinal sections [1] cone density in a retinal whole mount has not been analyzed. We found that cone density and presence appear normal in *Scn8a* ^dmu^ (Fig. 4) indicating that, like rods, cones are not morphologically different in *Scn8a* ^dmu^ mice.

## Discussion

We found a substantial loss in both a- and b-waves over a range of background and flash intensities in *Scn8a* ^dmu^ mice. This indicates that Na_v_1.6 is necessary for the retina to develop normal light responses to visual stimuli under a range of light adapting conditions. We investigated whether the reduction of retinal response in light adapting conditions is due solely to a photoreceptor defect or whether Na_v_1.6 may be present on cone bipolar cells, the loss of which could further reduce the b-wave. We envision three scenarios in which the a- and b-waves would be reduced in the absence of retinal Na_v_1.6 mice. Firstly, the effect could be solely due to a reduction in photoreceptor function. Secondly, the b-wave could be reduced due to the loss of Na_v_1.6 in cone bipolar cells while the a-wave reduction would be due to the loss of Na_v_1.6 in post-receptoral cells. Thirdly, the substantial reduction in the b-wave could be caused by a combination of the first two factors. A rigorous evaluation of these possibilities entails the intravitreal injection of mutant mice with pharmacologic agents. Attempts to do this have yielded inconsistent results due to the frailty of the mutants. Instead we used pharmacologic block of retinal signaling pathways in control littermates from *Scn8a* ^dmu^ litters to quantify the contribution of VGSCs to the light adapting b-wave in young mice as well as the proportion of the a-wave generated by the cones themselves.

The a-wave of the photopic ERG in mice has been previously shown to be sensitive to treatment with *cis*-2,3-piperidine-dicarboxylic acid (PDA), a non selective blocker of ionotropic glutamate receptors, indicating a post-receptoral component thought to be due to a combination of OFF bipolar cells and third order neurons [16], [18]–[20]. Attenuation of the a-wave at brighter backgrounds (0.9 log cd/m^2^ and up in *Scn8a* ^dmu^ mice is similar in magnitude to the loss of the a-wave following CNQX or CoCl_2_ in littermates. This could be caused by a total loss of the post-receptoral component of the a-wave in these mice or by a reduction in both components leaving a residual a-wave of similar amplitude to the photoreceptor isolated a-wave of controls but containing a post-receptoral component.

To further distinguish between these two possibilities we used TTX to acutely block VGSC function in wild-type control mice. We found no effect of TTX on the a-wave in agreement with previous results [11], [13], [21], [22], which suggests that the absence of Na_v_1.6 from retinal neurons is insufficient to cause the reduction in a-wave we see in *Scn8a* ^dmu^ mice. In addition the loss of b-wave amplitude we see in *Scn8a* ^dmu^ mice (∼70% over the brightest 6 backgrounds where the loss is most consistent) substantially exceeds the loss we see in the presence of TTX in controls (∼40% over the same backgrounds). This suggests that the primary cause of the reduction in b-wave amplitude in *Scn8a* ^dmu^ mice is a developmental defect in cone photoreceptor function similar to the effect observed in the rods [1].

Thus, based on our results we suggest that the most likely explanation for the reduction in the light adapted ERG of *Scn8a* ^dmu^ mice is a combination of a developmental photoreceptor defect in the cones that is equivalent to that of the rods and that the residual a-wave of the dmu mice contains both photoreceptor and post-receptoral components. It is possible that the b-wave of *Scn8a* ^dmu^ mice is reduced because of a change in the ratio of post-receptoral to photoreceptoral contributions to the a-wave - a decrease in the photoreceptoral component of roughly 20% over the 7 brightest backgrounds - masked by an equivalent increase in the post-receptoral component could generate the observed b-wave change. However it seems unlikely that a decrease in photoreceptor function should increase the amplitude of post-receptoral responses contributing to the a-wave while decreasing the b-wave. We were unable to propose a mechanism that could lead to the relatively large b-wave of *Scn8a* ^dmu^ mice at the dimmest background.

A subset of cone bipolar cells have voltage gated sodium currents [5], and Na_v_1.1, 1.2, and 1.6 have all been found in the outer plexiform layer by immuno-histochemistry [12]. However it is unknown what VGSC isoform(s) contribute to bipolar cell sodium current, an interesting question since each VGSC isoform has unique properties, which could be important in determining their function and susceptibility to neuromodulators. Na_v_1.6, in particular is unique amongst retinal VGSC isoforms in its ability to support persistent sodium current [23], [24] and its relative insensitivity to inhibition by phosphorylation [24]. Ichinose et al. [7] noted that the sodium currents they found in salamander bipolar cells were relatively sustained and slowly inactivating, consistent with persistent sodium currents. Our findings suggest that the dominant VGSC isoform in cone bipolar cell signaling may be Na_v_1.6 but a decisive proof, involving direct recording from bipolar cells in *Scn8a* ^dmu^ mice is outside of the scope of this report. Our results suggest that Na_v_1.6 may play a role in modifying signaling in cone bipolar cell signaling, consistent with the presence of Na_v_1.6 in the outer plexiform layer [12].

### Conclusions

In summary, we found that both the a- and b-waves were substantially reduced in *Scn8a* ^dmu^ mice under conditions of light adaptation. Analysis of the behavior of the ERG of wild-type mice subjected to various pharmacologic treatments leads us to suggest a model where the reduction of the ERG b-wave in *Scn8a* ^dmu^ is initiated by a defect in the physiology of the cone photoreceptors, augmented at higher luminance backgrounds by a loss of VGSC function in ON cone bipolar cells.

## References

[1] Côté PD, De Repentigny Y, Coupland SG, Schwab Y, Roux MJ (2005). Physiological maturation of photoreceptors depends on the voltage-gated sodium channel NaV1.6 (Scn8a).. J Neurosci.

[2] Swaroop A, Kim D, Forrest D (2010). Transcriptional regulation of photoreceptor development and homeostasis in the mammalian retina.. Nat Rev Neurosci.

[3] Holzhausen LC, Lewis AA, Cheong KK, Brockerhoff SE (2009). Differential role for synaptojanin 1 in rod and cone photoreceptors.. J Comp Neurol.

[4] Zenisek D, Henry D, Studholme K, Yazulla S, Matthews G (2001). Voltage-dependent sodium channels are expressed in nonspiking retinal bipolar neurons.. J Neurosci.

[5] Cui J, Pan ZH (2008). Two types of cone bipolar cells express voltage-gated na+ channels in the rat retina.. Vis Neurosci.

[6] Ichinose T, Lukasiewicz PD (2007). Ambient light regulates sodium channel activity to dynamically control retinal signaling.. J Neurosci.

[7] Ichinose T, Shields CR, Lukasiewicz PD (2005). Sodium channels in transient retinal bipolar cells enhance visual responses in ganglion cells.. J Neurosci.

[8] Ohkuma M, Kawai F, Horiguchi M, Miyachi E (2007). Patch-clamp recording of human retinal photoreceptors and bipolar cells.. Photochem Photobiol.

[9] Pan ZH, Hu HJ (2000). Voltage-dependent na(+) currents in mammalian retinal cone bipolar cells.. J Neurophysiol.

[10] Bui B, Fortune B (2004). Ganglion cell contributions to the rat full-field electroretinogram.. J Physiol.

[11] Miura G, Wang MH, Ivers KM, Frishman LJ (2009). Retinal pathway origins of the pattern ERG of the mouse.. Exp Eye Res.

[12] Mojumder DK, Frishman LJ, Otteson DC, Sherry DM (2007). Voltage-gated sodium channel alpha-subunits na(v)1.1, na(v)1.2, and na(v)1.6 in the distal mammalian retina.. Mol Vis.

[13] Mojumder DK, Sherry DM, Frishman LJ (2008). Contribution of voltage-gated sodium channels to the b-wave of the mammalian flash electroretinogram.. J Physiol.

[14] Viswanathan S, Frishman LJ, Robson JG, Harwerth RS, Smith EL (1999). The photopic negative response of the macaque electroretinogram: Reduction by experimental glaucoma.. Invest Ophthalmol Vis Sci.

[15] De Repentigny Y, Côté PD, Pool M, Bernier G, Girard S (2001). Pathological and genetic analysis of the degenerating muscle (dmu) mouse: A new allele of Scn8a.. Hum Mol Genet.

[16] Sharma S, Ball SL, Peachey NS (2005). Pharmacological studies of the mouse cone electroretinogram.. Vis Neurosci.

[17] Dang TM, Tsai TI, Vingrys AJ, Bui BV (2011). Post-receptoral contributions to the rat scotopic electroretinogram a-wave.. Doc Ophthalmo Advances in Ophthalmology.

[18] Shirato S, Maeda H, Miura G, Frishman LJ (2008). Postreceptoral contributions to the light-adapted ERG of mice lacking b-waves.. Exp Eye Res.

[19] Bush RA, Sieving PA (1994). A proximal retinal component in the primate photopic ERG a-wave.. Invest Ophthalmol Vis Sci.

[20] Robson JG, Saszik SM, Ahmed J, Frishman LJ (2003). Rod and cone contributions to the a-wave of the electroretinogram of the macaque.. J Physiol.

[21] Kurimoto Y, Kondo M, Ueno S, Sakai T, Machida S (2009). Asymmetry of focal macular photopic negative responses (PhNRs) in monkeys.. Exp Eye Res.

[22] Popova E, Kupenova P (2010). Contribution of voltage-gated sodium channels to b- and d-waves of frog electroretinogram under different conditions of light adaptation.. Vision Res.

[23] Van Wart A, Matthews G (2006). Impaired firing and cell-specific compensation in neurons lacking nav1.6 sodium channels.. J Neurosci.

[24] Chen Y, Yu FH, Sharp EM, Beacham D, Scheuer T (2008). Functional properties and differential neuromodulation of Na(v)1.6 channels.. Mol Cell Neurosci.

